# A novel minimally invasive and press-fit method for symphysiodesis — a biomechanical analysis

**DOI:** 10.1186/s40634-023-00660-6

**Published:** 2023-09-28

**Authors:** Tobias Fritz, Marcel Orth, Sascha J. Hopp, Jeremy Briem, Jill Hahner, David Osche, Tim Pohlemann, Antonius Pizanis

**Affiliations:** 1 Department for Trauma, Hand and Reconstructive Surgery, Saarland University Medical Center, Kirrbergerstr. 1, Homburg, Saarland 66421 Germany; 2 Lutrina Hospital, Kaiserslautern, Brüsseler Str. 7, 67655 Kaiserslautern, Germany; 3 Department of Trauma and Reconstructive Surgery, BG Trauma Center Tuebingen, Eberhard Karls University Tuebingen, 72076 Tuebingen, Germany

**Keywords:** Internal fixator, Pubic symphysis, Symphysis, Symphysiodesis, Arthrodesis symphysis, Bone transplant

## Abstract

**Purpose:**

Does the cylindrical shaped bone block allow a stable construct for the arthrodesis of the pubic symphysis compared to a rectangular shaped bone block. The cylindrical shaped bone block stabilized by a 3.5 symphyseal plate is inferior to the stabilization with an internal fixator.

**Methods:**

This study analyzed the arthrodesis of the pubic symphysis on 24 synthetic pelvises, using a rectangular shaped bone block (control group) or a cylindrical shaped bone block, stabilized with a symphysis locking plate (*n* = 8) as the standard clinical procedure. Additionally we analyzed the stability using an internal fixator.

**Results:**

This study showed that utilizing a cylindrical shaped synthetic bone graft results in a significant higher contact area and compression force compared to the classical rectangular shaped graft. Furthermore, the stabilization with an internal fixator had the tendency for increases of compression force and contact area, yet without a statistical significance, when compared to the plate fixation.

**Conclusion:**

The novel method of cylindrical symphysis resection and cylindrical bone block implantation allowed an increased biomechanical stability compared to using a classical rectangular bone graft, also resulting in higher contact area. Moreover, this technique would also allow a minimally invasive approach for this purpose, which in turn could preserve perisymphyseal ligaments, thereby improving healing in a clinical context.

## Introduction

The instability of the pubic symphysis can result from a traumatic rupture, but also results from non-traumatic conditions like osteitis pubis, sportive overuse with repeated micro tears, rheumatologic disorders or postpartal after delivery. Persisting instability of the pubic symphysis can result in reduced mobility and poor patient outcomes. The treatment of these patients is often challenging and in some cases, a fusion of the pubic symphysis (symphysiodesis) is the only alternative to improve the outcome.

The gold standard for a fusion of the symphysis is either one plate in cranial position or double plating with an additive “bumper” plate in 90°, in frontal plane of the symphysis [5]. Complications such as nerve irritation which could lead to a chronic pain syndrome, infection and also induced necrosis of the soft tissue, especially in the growing population of obese patients [9–12]. On top of the mentioned complications above, this procedure may result in delayed bone healing or even absence of consolidation of the bone graft.

In a previous study we published the feasibility of a novel minimally-invasive press-fit method using a cylindrical bone graft for fusion. Using this method, the surgical approach could be minimalized by restricting the resection solely to the cranial ligaments of the pubic symphysis [1] and only the discus and the sclerotic parts of the pubic symphysis need to be removed until vital cancellous bone can be identified. The autologous bone graft, which is harvested from the iliac crest or the proximal tibia, will be transplanted into this area. Although the surgical technique has been shown to be feasible, a quantitative biomechanical analysis of the minimally-invasive graft transplantation technique can provide additional information regarding the biomechanical implications of this surgery.

In this study we analyzed the biomechanical capabilities of a cylindric bone graft and a rectangular bone graft with the use of either a 3.5 symphysis locking dynamic compression plate (SLDCP) (Synthes, Malans, Switzerland) or the stabilization using an internal fixator (USS™-Fracture®, Synthes, Oberkirch, Switzerland) to establish a truely novel minimally invasive technique [4, 8]. In a clinical setting, using only a small incision to transplant a cylindrical autologous bone graft and insert the Schanz screws, fracture clamps, and fracture rod allows a minimally invasive symphysiodesis. The surgical technique for the internal fixator has already been adapted to open book injury of the pubic symphysis in a case report and a biomechanical study published previously [3, 4].

The aim of this study was to investigate the biomechanical capabilities of a cylindrical bone graft stabilized by a 3.5 SLDCP or an internal fixator compared to the commonly used rectangular one stabilized by a 3.5 SLDCP. Furthermore, we compared the cylindrical bone graft stabilized by an internal fixator to allow a truly minimally invasive surgical technique.

We hypothized that the cylindrical bone graft will increase the contact area to the symphyseal bone and using the internal fixator an equivalent stability will be achieved, compared to plate osteosynthesis.

## Methods

### Specimen

For this study anatomical composite pelvises, solid foam, dense cancellous bone, consisting of both iliac wings and the sacrum, of regular dimensions were used (width: 305 mm; height: 160 mm) (Model 4060®, Synbone, Switzerland).

### Sensors

The 3D-motion of the pelvis was measured by an optical tracking system consisting of 4 cameras (Prime 13®, Optitrack, USA). The optical system detects optical markers with a resolution of 1280 × 1024 Pixel, which allows an accuracy of 0.2 mm, as stated by the manufacturer. We attached 6 optical markers at each pelvic half using a standardized template. The system specific software (Motive 2.1®, Optitrack, USA) was used to process the measurements into a respective 3D coordinate system. Mathematical calculations specified the relative movement of the main fragments in three spatial planes. The maximum displacement was defined as maximum distance at loading.

As shown in previous studies [4, 16] compression forces and contact area of the symphysis could be measured dynamically with a thin electro-sensitive sensor film and monitored by I-scan® software (Tekscan Inc., USA). To provide high accuracy and repeatability, a new sensor was used on each series of experiments in order to reduce the effect of sensor deterioration [4, 15, 16].

The sensor films used in this study were of the type 5033 (Tekscan Inc., USA), with a matrix height of 38.4 mm and matrix width 26.4 mm. The manufacturer provided a density of 144.1 sensels/cm^2^. All sensors were conditioned before initial testing according to the manufacturers’ recommendations by loading 2 layers of rubber-covered aluminum plates to 120% of the expected maximum (400 N) for 5 loading cycles in a universal material testing machine (Instron E10000, Instron, USA), in order to allow the sensor film to get used to the pressure, and even more so that no data will be lost [14]. Thereafter, software-supported 2-point calibration was performed by loading the sensor at 50 and 500 N.

### Segmental pressure mapping

Using the I-scan software, the intrasymphyseal grafts were divided into 3 segments (equal thirds of the complete sensor film area). The segments were labelled cranial, intermediate, and caudal segment. This data was later analyzed to detect pressure in the 3 segmented areas [4, 15].

### Experimental setup

**Fig. 1:**
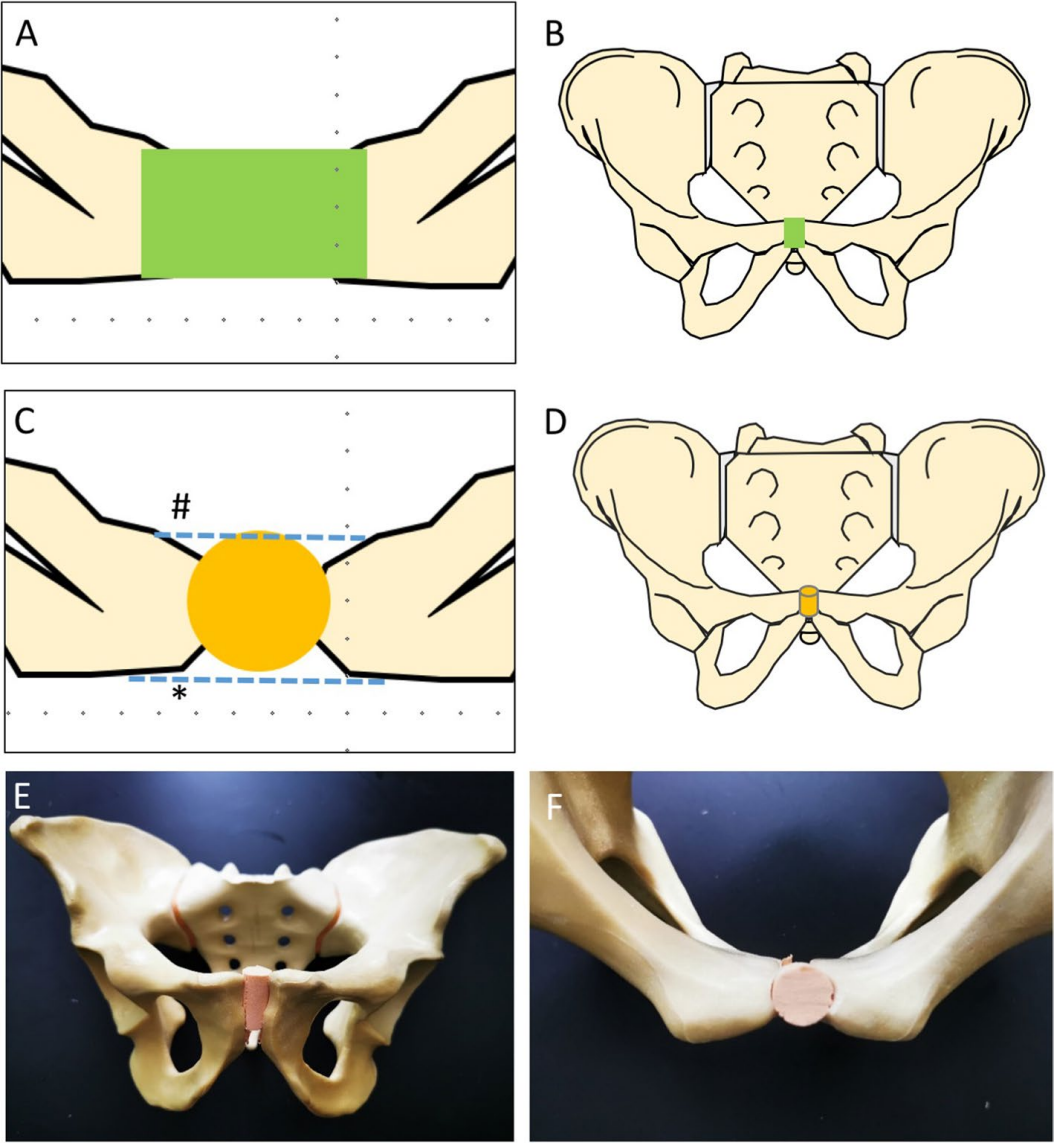
Schematic drawing of the synthetic bone graft methods analyzed in this study. **A**, **B**: Drawing of the Control group using a rectangular shaped bone graft (green) **C**, **D**: Schematic drawing of the experimental groups using a cylindrical shaped bone graft (yellow), which would leave the anterior (*) and posterior (#) ligaments of the pubic symphysis intact. **E**, **F** Implantation of the cylindrical shaped bone graft in a synthetic pelvis (Synbone, 4060, Malans, Switzerland)

In this study we investigated the following three groups, each consisting of *n* = 8 pelvises:

SLDCP Control Group (Fig. 1A & B): A rectangular bone area of the symphysis was resected using an oscillating saw on a standardized template (width 12 mm; height 30 mm). After that, an artificial bone block of the same size was transplanted into the gap. Whilst transplanting the graft, a sensor film was placed between the bone block and the pubic symphysis.

SLDCP Group and Internal Fixator Group (Fig. 1 C, D, E, F): A cylindrical synthetic bone graft was resected using a surgical diamond trephine instrument (12.6 mm diameter; 30 mm height) (SDI, boneArtis AG, Switzerland). After resection, the synthetic cylindrical bone graft (15.4 mm diameter; 30 mm height) was impacted.

After implantation of the synthetic bone grafts, a reduction clamp was placed and the pubic symphysis was compressed with 50 N. Thereafter the pubic symphysis was stabilized as described below.

### Stabilization using 3.5 mm SLDCP (Control Group and SLDCP Group)

According to our previous studies, we followed the same protocol [4].

- Step I: placement of the plate across the pubic symphysis and placement of both medial cortical screws to achieve dynamic compression [4].
- Step II: Finalizing the osteosynthesis by placing the interlocking lateral screws. A torque control for the interlocking screws was used (PSR 960, 3 Nm, Bosch, Germany). The initially used reduction tong was removed [4].

### Stabilization using the internal fixator (IF Group)

A 5 mm transpedicular Schanz screw (Synthes, Germany) was implanted on both sides of the pelvis, parallel to the pubic symphysis (cranial to caudal). Two fracture clamps were inserted along these Schanz-screws. After the fixation rod of 35 mm length and 6 mm diameter (Synthes, Germany) was pushed between these two fracture clamps, and the following stabilization steps were applied [4].

- Step I*:* The socket wrench (Ø 11 mm) was placed on both Schanz screws and then tilted laterally [4]. Afterwards, the clamps/Schanz screws were secured in this position. This maneuver resulted in caudal compression [4].
- Step II: The fracture clamps were slid medially and a Ø 6 mm socket wrench was used to fix the fracture clamps on the vertical rods. Further steps were performed as in the other groups. As used in spine surgery, this technique can clinically be performed in a minimally invasive approach [4].

Sensor films were placed in the symphysis and the symphyseal gap was reduced with a tong (Synthes, Umkirch, Germany) that was applied at prepared spots on both sides of the pubic symphysis, until a defined compression force of 50 N was obtained [4, 15]. Corresponding forces inside the pubic symphysis and the contact area were measured at the end of each step by I-scan® sensor films (TekScan, USA) [4].

### Each pelvis was mounted into a universal testing machine (Instron E10000, Instron, USA)

**Fig. 2:**
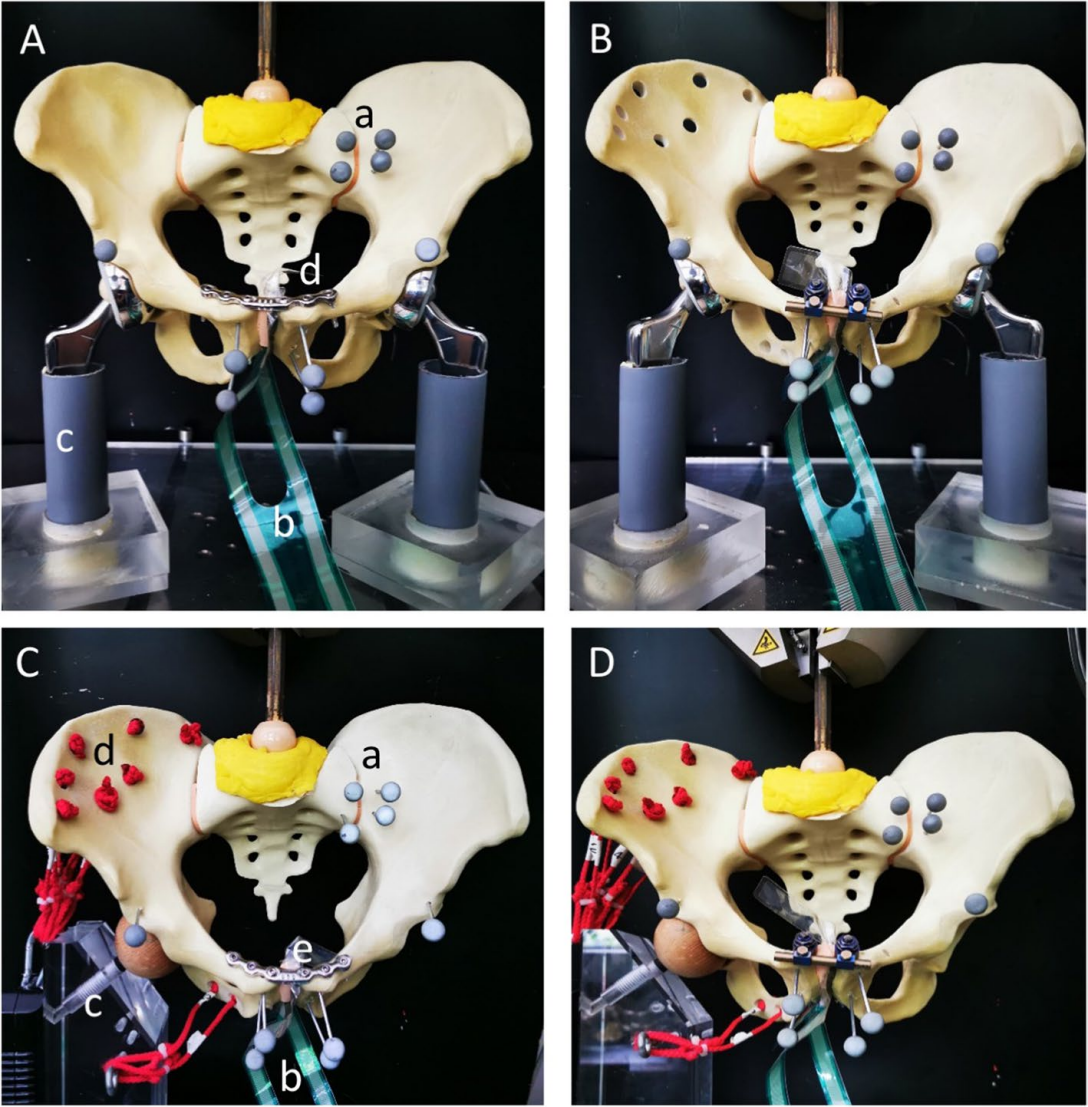
**A** Shows the experimental setup of the SLDCP group (**A**) and Internal Fixator group (**B**), using the bilateral leg stance biomechanical model a) optical markers, b) sensor film, c) bipolar prosthesis, d) osteosynthesis (**C**) Experimental setup of the SLDCP group and Internal Fixator group (**D**), using the unilateral leg stance biomechanical model, a) optical markers, b) sensor film, c) unilateral femoral head, d) wires to simulate muscle strains, e) osteosynthesis

To simulate biomechanical loading all pelvises were tested in a single leg stance and a bilateral leg stance, using a custom-made mount as in previous studies (Fig. 2 A-D) [2, 4]. Additionally one 7.3 mm sacroiliac screw (Synthes, Germany) was placed in the right S2 vertebra, to add stability to the sacroiliac-joint, which is needed for the test-set up.

All pelvises were axially loaded using a ceramic hip prosthesis head (28 mm, Zimmer Inc., Warsaw, Indiana, USA) articulating at 45° to the proximal sacrum in a custom-made fitting cement cup (Technovit 3040®, Heraeus Kulzer GmbH, Germany) on the first sacral vertebra [4].

All pelvises were positioned with a preload of 50N, whereby a 25° inclination and 15° abduction were set, reflecting the vector of the maximum load during a gait cycle shortly before foot lifting [4]. The respective maximum load and relief plateau was held for 10 s each. The first two loads were defined as setting cycles. During the following 5 cycles the dislocation and was measured. The force was reduced to the 50N initial force and increased by 50N steps up to 400N. The 400N were calculated following the presumed mean weight of a person being 80 kg an each of the lower extremities weighing 20 kg. This results in a residual weight of 40 kg resting on the pelvis. Therefore, the force acting on the pelvis was assumed to be approximately 400 N. For the single leg stance also 400 N axial loading was used, due to decreased stability of the posterior pelvic ring of the pelvis models used in this study. The 50N steps in between represent the gradual increase. After the final cycle all pelvises were removed from the universal testing machine and the force and contact area inside the pubic symphysis were recorded (described as FINAL in the figures). Then the pelvises were placed from the single leg stance to a bilateral stance and the measurements were repeated.

### Data acquisition, processing and statistical analysis

After each reduction and after every step, compressive forces and the contact area were measured with I-scan® sensor films 5033 (TekScan, USA). In addition, the contact areas of the cranial, central, and caudal thirds of the symphysis (contact distribution in %) were assessed with I-scan software 6.02 (TekScan, USA) to represent an indicator for reduction quality [4]. The spatial instability and movement were acquired by an optical tracking system (Prime 13®, Optitrack, USA). The optical markers were mounted at standardized positions on each pelvis using a template for positioning. The analysis was performed using the software provided by the manufacturer (Motive 2.1®, Optitrack, USA).

The data was processed by Sigma Plot 13.0 (Systat, Germany), using descriptive statistics consisting of mean, range, and quartiles (25–75%). Differences to the stabilization were analyzed using an ANOVA Repeated Measures with a Dunnett’s post hoc test (Post stabilization was set as control in each group) [4]. Differences between groups were analyzed with Kruskal–Wallis analysis of variance (ANOVA) on ranks with post hoc Student–Newman–Keuls test. A *p* value < 0.05 was defined to indicate significant differences [4].

## Results

### Effect on the intrasymphyseal compression force – unilateral leg stance

**Fig. 3:**
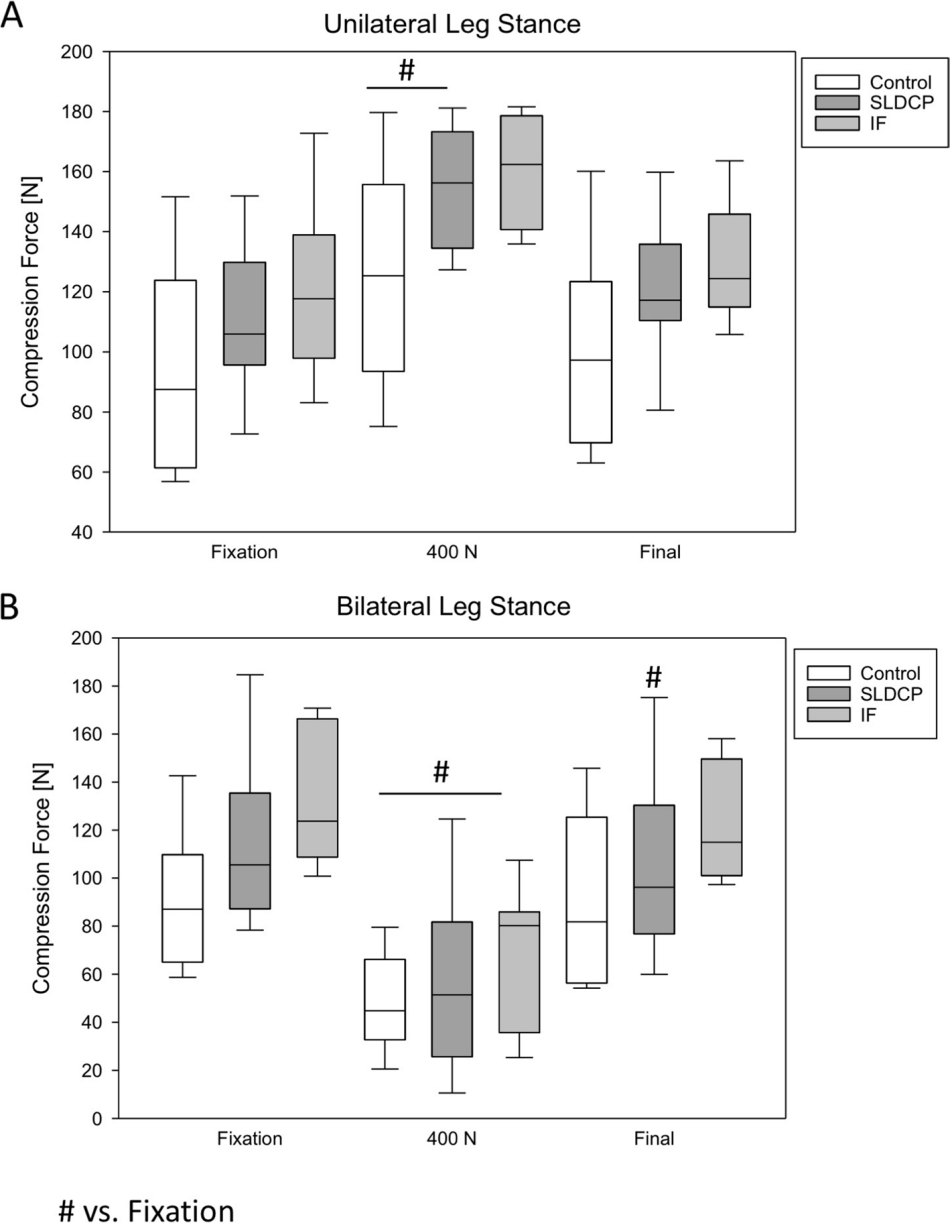
Compression forces on the grafts during axial loading. * *p* < 0.05 vs. rectangular shaped bone graft (Control Group); # *p* < 0.05 vs. fixation in each group. SLDCP, symphyseal locking dynamic compression plate; IF, Internal Fixator

Under axial loading up to 400N, a significant increase of the compression force was found in both the Control and the SLDCP group compared to post stabilization. However, the highest compression force was achieved in the IF Group (Fig. 3A) yet without significant difference to the other groups (Fig. 3A).

### Effect on the intrasymphyseal compression force – bilateral leg stance

Under axial loading in bilateral stance, the compression force significantly decreased in all groups (Fig. 3B). Of interest, the IF Group still showed the highest compression force compared to the other groups (Fig. 3B). After loading a significant loss compared to the state after stabilization was found for the group with cylinder and SLDCP. In the other groups, no significant differences compared to fixation were found.

No differences between the groups were detected with respect to the compression force inside the pubic symphysis (Fig. 3B).

### Effect on the intrasymphyseal contact area – unilateral leg stance

**Fig. 4:**
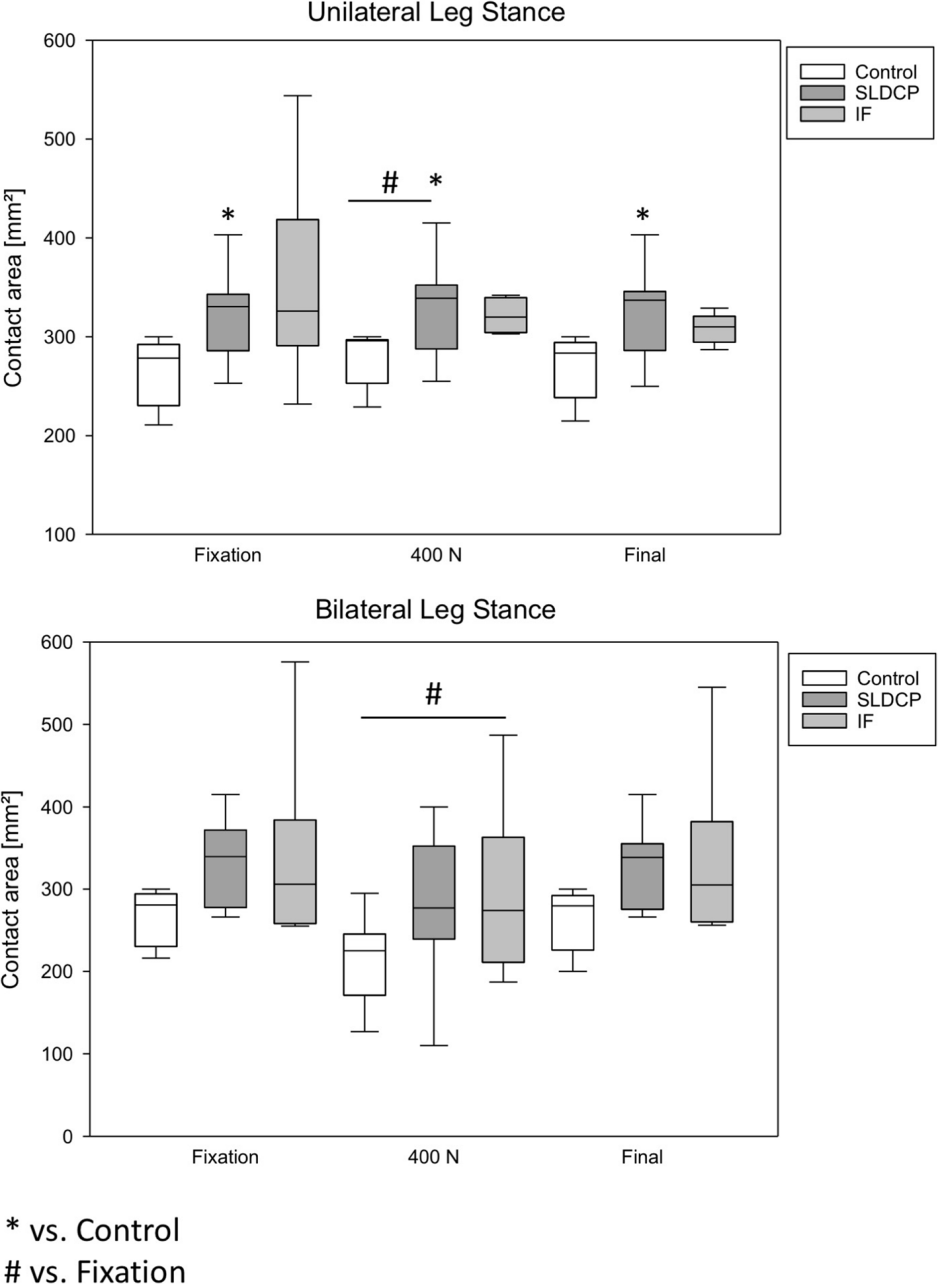
Contact area of the grafts during axial loading. * *p* < 0.05 vs. rectangular shaped bone graft (Control Group); # *p* < 0.05 vs. fixation in each group. SLDCP, symphyseal locking dynamic compression plate; IF, Internal Fixator

The SLDCP group showed a significant higher contact area compared to the control group at all-time points, while the internal fixator showed no difference compared to the other groups (Fig. 4A). Under axial loading of 400 N both SLDCP groups showed a significant increase of the contact area, while the internal fixator having the highest amount of contact area did not increase further. After testing, all groups reached their initial contact area.

### Effect on the intrasymphyseal contact area – bilateral leg stance

No significant differences of the contact area between the groups were detected (Fig. 4B). Under axial loading of 400N the contact area significantly decreased in all groups (Figs. 4B). After testing, the values of their initial contact area were reached in all groups.

### Effect on the intrasymphyseal segmental compression force – single leg stance and bilateral leg stance

**Fig. 5:**
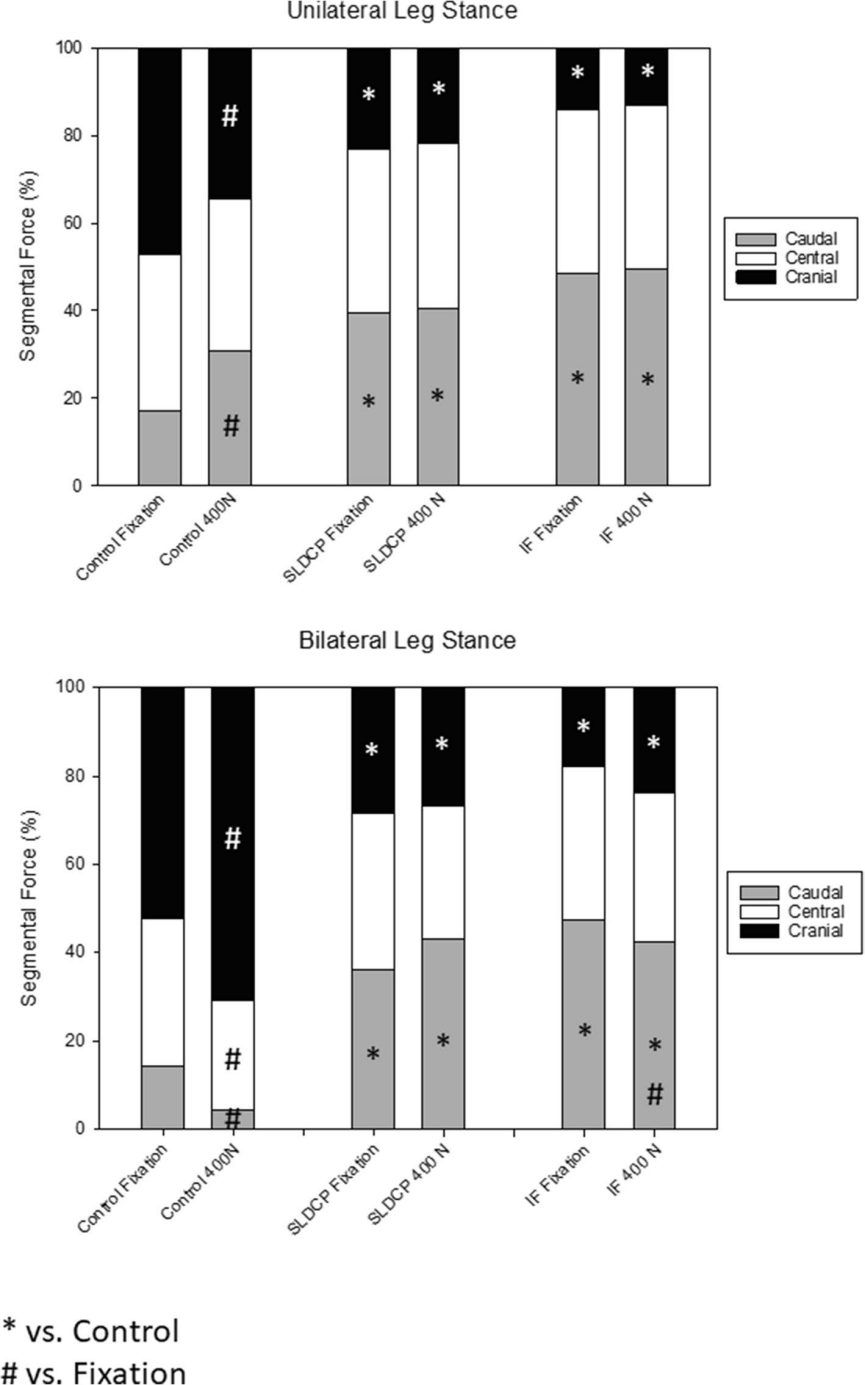
Percentage distribution of the intrasymphyseal compression force in unilateral (**A**) and bilateral stance (**B**). * *p* < 0.05 vs. rectangular shaped bone graft (Control Group); # *p* < 0.05 vs. fixation in each group. SLDCP, symphyseal locking dynamic compression plate; IF, Internal Fixator

Compared to the rectangular synthetic bone graft, the cylindrical one produced a significantly lower compression force in the cranial area, but in contrast allowed a significantly higher contact in the caudal area Fig. 5A, B. During axial loading, the compression force of the rectangular synthetic bone graft increased in the cranial area significantly and decreased in the caudal area significantly Fig. 5A, B. In the cylindrical graft groups, no changes were detected. In the cylindrical synthetic bone graft groups, the stabilization using a SLDCP resulted without changes, the internal fixator also decreased significant caudally Fig. 5A, B.

### Three-dimensional movement of the pubic symphysis under loading

The vertical and rotational instability measured by the 3D optical system showed no significant difference between the test groups. There were no significant differences in the movement, since all implants could maintain the stability of the pelvic ring (Table 1).

**Table 1:** The data is given in mm and shows the Δ after the osteosynthesis compared to 400N axial loading. No significant differences were detected

Δ Osteosynthesis/400N	Control	SLDCP	IF
**Single leg stance**			
Symphysis cranial	-0.12 SD 0.04	-0.12 SD 0.09	-0.2 SD 0.12
Symphysis caudal	1.16 SD 1.28	0.33 SD 0.23	0.24 SD 0.06
Iliosacral joint	-0.42 SD 0.14	-0.67 SD 0.31	-0.8 SD 0.29
**Bilateral stance**			
Symphysis cranial	-0.005 SD 0.04	0.04 SD 0.14	0.08 SD 0.37
Symphysis caudal	-0.16 SD 0.09	-0.30 SD 0.29	-0.56 SD 0.44
Iliosacral joint	-0.001 SD 0.6	0.23 SD 0.17	0.12 SD 0.5

## Discussion

The results of the present study indicate that the usage of a cylindrical bone graft allows the same biomechanical capabilities as the gold standard rectangular bone graft and that the application of an internal fixator for stabilization of the symphysis may be beneficial when compared to a common plate fixation.

Chronic instability of the pubic symphysis often results in chronic pain with reduced mobility of the patients. When conservative treatment fails, the arthrodesis of the pubic symphysis (symphysiodesis) is considered to be the gold standard to achieve adequate stability and pain relief [1, 8]. According to the literature [6, 9], a tricortical bone block from the iliac crest of the synthetic pelvis served as control group in the present study. A cylindrical graft, which in a clinical setup could be harvested from the proximal tibia, as described in our previous study [9] was used in the experimental groups in order to improve the cancellous bone contact and allow the possibility of introducing it by minimal invasive surgery. The usage of press-fit cylinders in orthopaedics has been well-established in recent years [2, 6]. However, its use in the treatment of pelvic non-unions in combination with a minimal invasive surgical technique has been described only in very few cases [9].

The stabilization of the pubic symphysis for the arthrodesis is usually performed with a cranial 3.5 symphyseal locking plate (SLDCP). Some authors recommend a second anterior “bumper” plate [6, 9, 12]. However, no further biomechanical studies have been published to evaluate the advantage of this extended stabilization. Although the surgical technique has been described over 20 years ago, only few specialists treat a little number of cases per year [14] and the need of a second “bumper” plate for adding stability or securing the strut graft still remains unclear. In this study, the cranial 3.5 SLDCP was used for the control group with a rectangular synthetic bone block and for the experimental group using a cylindrical synthetic bone graft. In previous studies, the usage of an internal fixator utilized for spine surgery, provides increased stability compared to current plate stabilization techniques of the pubic symphysis [5, 14]. The combination of both surgical techniques would permit a truly minimally invasive technique to perform the symphysiodesis.

To investigate the biomechanical stability, the bilateral stance model and the single leg model were used [2, 5]. The bilateral stance model is necessary for the analysis of the horizontal movement of the anterior pelvic ring. On the other hand, this model is of limited use when examining combined vertical and horizontal forces of the pelvis. For this purpose, the single-leg stance model is beneficial. The synthetic bone models are commonly used for biomechanical studies [5]. The anatomical models which were used in this study consisted of a solid polyurethane foam and a harder shell and simulate a dense cortex. Although the manufacturer claims that the biomechanical properties are less compared to natural bone, composite pelvises provide standardized dimensions, which allows for small sample sizes [7]. Also, previous studies showed that the symphyses of the composite pelvises used in this biomechanical analysis are similar to the dimensions of human specimen [7]. However, composite pelvises lack the soft tissue connections, and it remains unclear how much this would affect movements of a defined pubic area in reality. Although daily activity results in high axial forces, the stability of the posterior pelvic ring of artificial pelvises is limited to 400 N, which is why most studies use 400 N as a maximum loading. Under axial loading, no significant changes were detected for 3D movement, which allows the conclusion that all fixation techniques resulted in a reliable primary stability of the symphysiodesis.

The compression forces and the contact area were measured as published in previous studies [4, 14, 15]. Our results showed that a sufficient stabilization was obtained in all groups. The bilateral leg stance model resulted in a decreased compression force and contact area, whilst the single leg stance model resulted in an increased compression force and contact area. The initial significant higher compression force of the internal fixator could not be obtained during axial loading. Interestingly, the contact area was maintained during the course of loading when the internal fixator was used and the internal fixator showed the highest compression force of the investigated groups. The rectangular and cylindrical synthetic bone graft showed no significant difference of the contact area. Regarding the contact area as an indicator of good reduction and expected increased healing potential, all groups showed satisfying results. Analyzing the segmental force distribution, the rectangular graft groups had a caudalization in the single leg stance and cranialization in the bilateral stance model, while the segmental force distribution of the cylindrical shaped synthetic bone grafts only changed slightly and thereby behaved more constant.

Further analysis revealed even higher distribution of the contact area across the symphysis when the fixation was done with internal fixator. In contrast, when plates were used for stabilization, clear loss of caudal contact area occurred after loading in the bilateral leg stance model. The internal fixator was placed parallel to the pubic symphysis and, thereby a more homogenous distribution of compressive forces from the implant to the symphysiodesis could result. Morris et al. described that no implant is capable of carrying physiological loads forever, but a stable biomechanical environment is of fundamental importance for healing [13]. Therefore, the results of the present study indicate that the usage of a cylindrical bone graft allows the same biomechanical capabilities as the gold standard rectangular bone graft and that the application of an internal fixator for stabilization of the symphysis may be beneficial when compared to a common plate fixation. Although no significant differences were detected between the groups under axial loading, the internal fixator tended to show higher compression force in this study.

## Limitations

This study did not use cycled loading and long-term fatigue measurement; therefore, no conclusion about hardware loosening or breakage in the course of long-term use can be derived.. Also the anatomical models which were used in this study, lack of soft tissue and only simulate human bone, hence, further studies with cadaveric pelvises are required to assess higher loading forces under anatomically more realistic conditions in order to investigate the stability of the pelvis with the attached ligaments.

## Conclusion

The surgical gold standard for the treatment by symphysiodesis is extensive and bears a high risk for complications. The use of a cylindrical graft and an internal fixator allows a minimally invasive surgical technique. This study shows that the contact area and the compression force on the grafts with a new technique are at least equivalent to the current golden standard, thereby supporting the minimal invasive surgery method. Based on these results, a defined in-vivo clinical application study of this minimally invasive technique using a cylindrical bone graft in combination with an internal fixator would be of considerable interest to achieve for patients with chronic symphyseal instability and pain.

## Abbreviations

AO: Arbeitsgemeinschaft Osteosynthese
DCP: Dynamic compression plate
e.g.: Exempli gratia
HOMFOR: Homburg Funding (Funding by Saarland University)
kHz: Kilohertz
mm: Millimeter
N: Newton
Nm: Newtonmeter
SLDCP: Symphyseal locking dynamic compression plate
3D: Three-dimensional
